# Microsurgical endodontic treatment of the upper molar teeth and their relationship with the maxillary sinus: a retrospective multicentric clinical study

**DOI:** 10.1186/s12903-021-01610-3

**Published:** 2021-05-12

**Authors:** S. Taschieri, B. Morandi, M. Giovarruscio, L. Francetti, A. Russillo, S. Corbella

**Affiliations:** 1 Department of Biomedical, Surgical and Dental Sciences, Università Degli Studi Di Milano, 20123 Milan, Italy; 2 Institute of Dentistry, I. M. Sechenov First Moscow State Medical University, Moscow, Russia; 3 Department of Endodontics, Faculty of Dentistry, Oral and Craniofacial Sciences, King’s College London, London, UK; 4 IRCCS Istituto Ortopedico Galeazzi, Via R. Galeazzi 4, 20161 Milan, Italy; 5 Maxillofacial and Dental Unit, Fondazione IRCCS Cà Granda Ospedale Maggiore Policlinico, Via Commenda 10, 20122 Milan, Italy

**Keywords:** Microsurgical endodontics, Endodontic surgery, Apical surgery, Maxillary sinus, Maxillary molars, Postoperative pain and swelling

## Abstract

**Purpose:**

To assess the clinical and radiographic success rate of microsurgical endodontic treatment of upper molar teeth in relationship with the maxillary sinus, with 12 months follow-up.

**Methods:**

Patients treated with microsurgical endodontic treatment of upper molar teeth in the period between 2017 and 2019 were recruited from two dental clinics according to specific selection criteria. The outcomes were determined based on clinical and radiographic results taken three, six and 12 months post-operatively, compared with those taken immediately before and after surgery. Clinical and radiographic outcomes were recorded. The distance between the most apical part of the root and of the lesion to the maxillary sinus was measured on CBCT images before the surgery. Patient-related outcomes were recorded.

**Results:**

Out of 35 patients evaluated, 21 were selected according with the selection criteria for a total of 27 roots and 29 canals treated. After 12 months, 18 patients showed a complete healing whereas three demonstrated incomplete healing. Consequently, the success rate in this study was 85.7% after one year. In 28.5% (6 patients) there was a perforation of the Schneiderian membrane that didn’t seem to affect the outcome. All patients kept the molar one year later. The pain level decreased significantly over the time during the first week after surgery.

**Conclusion:**

Microsurgical Endodontic treatment of the upper molar teeth should be considered a valid and predictable treatment option even in case of Schneiderian membrane perforation. Future clinical studies with a larger sample size are needed to compare the results obtained.

## Background

Endodontic surgery is a surgical technique for the maintenance of devitalized teeth with apical pathology after failed endodontic therapy or when nonsurgical treatment is not possible or not recommended [1, 2].

The aim of this surgical technique is to eliminate the apical lesion and at the same time trapping the interradicular infection by sealing the root-end after resecting the last three millimetres of its apical portion [3].

In upper maxillary molar teeth, the endodontic surgery is complicated by the difficulty of accessing the surgical area. In addition, the anatomical complexity of the canal systems that characterise upper jaw molars and the relationships between them and the maxillary sinus could increase the difficulty of surgery [4, 5].

With regarding of the maxillary sinus, it is a bony cavity with a pyramidal structure involving the lateral wall of the nose, the base of the orbit and the zygomatic bone. It appears to be the largest of the paranasal sinuses. This bony cavity is characterised by the presence of the ostium located in the upper part of the medial wall, acts as an overflow drain. Ostium represent the opening of the nasal cavity, located below the middle nasal concha [6, 7]. The maxillary sinus is lined by a ciliary epithelium containing beaker cells that transports mucus with possible presence of bacteria and eventually foreign material introduced toward the maxillary ostium [8].

Some anatomical features of the the maxillary sinus, such as the presence of bony septa and of the alveolar antral artery, can influence and complicate the surgical procedure, and should be considered carefully before any surgical intervention in the region [9–14].

The aims of the maxillary sinus are to lighten the skull, be a space for conditioning breathable air, be a resonance chamber for the human voice and be an immunological barrier [7].

Many different radiographic and cadaveric studies have shown the relationship between the root apices of the maxillary posterior teeth, the periapical lesion, if present, and the sinus floor [15–23]. For example, Eberhardt et al. [23] reported a mean distance of 1.97 mm between the posterior maxillary teeth and the maxillary sinus floor and Von Arx et al. [24] demonstrated greater proximity between the maxillary sinus floor and periapical lesions as compared to maxillary sinus floor and the apices of the roots and this should warn the surgeon during the removal of the lesion.

Whereas the margin of the lesion and the floor of the maxillary sinus is not clear in two-dimensional images, current guidelines suggest the use of Cone Beam Computed Tomography (CBCT) for preoperative diagnosis and treatment. Radiographic studies have demonstrated the benefit of CBCT in detecting the size, presence, extension and location of the periapical lesion, as well as nearby anatomical structures [25–29].

Considering the frequent proximity of the maxillary sinus, endodontic surgery of upper premolars and molars can produce an accidental oroantral communication (OAC). OACs can occur during various stages of surgery, for example during bone or lesion removal or during root-end resection, and then, it can cause acute or chronic maxillary sinusitis, which usually are consequences of the displacement of bacteria from infected periapical tissue, the resected root tips or the bone drilling dust in the sinus [21, 30–35]. Ericson et al. [34] performed periapical surgery in 159 premolars and maxillary molars, creating OAC in 18% of the cases. According to these authors, the symptoms of maxillary sinusitis with thickening of the sinus membrane can be caused by the introduction of foreign bodies into the sinus during surgery. Other studies have found that the type of biofilm and the correlation between the bacteria present in the pathological dental elements communicating with the sinus and those present in the infected sinus, determine the severity and the spread of the infection [36]. Jerome and Hill [37] suggested the use of gauze to prevent the penetration of foreign bodies. Hauman et al. [38] stated that the sinus mucosa, complete with cilia, regenerates about five months after its surgical removal and therefore the invasion of the maxillary sinus does not appear to cause a permanent alteration of either the sinus membrane or its physiological function and the sinusitis will be resolved once adequate ventilation is restored. It is interesting to note that a recent study has shown that the perforation without repair of the sinus membrane during sinus lift procedure, did not affect the result of bone grafting and implant survival rate [39]. Few studies have focused on apicectomies of posterior teeth whose success rate ranged from 44 to 88% [21, 40] depending on the surgical technique and on the differences in the postoperative evaluation criteria.

Studies on surgical endodontics of upper molars have the function of validate a pre-clinical and surgical evaluation procedure as standardized as possible that can offer a valid therapeutic alternative to tooth extraction.

Within the limitation of the study, the primary aim was to evaluate clinical and radiographic success rate of apicectomies in upper molar teeth. The secondary outcome was to evaluate the relationship between the apex and the periapical lesion to the sinus floor as well as the presence of an oroantral communication. Finally, the quality of life of the patients were analyzed. Follow-up examinations to evaluate the outcomes were performed for 12 months.

## Methods

For this retrospective multicentric study, patients who have been treated with microsurgical endodontic procedure of the upper molar teeth in the period between 2017 and 2019 were recruited from two different dental clinical practices.

The study population was composed of patients from the Dental Clinic of the Department of Biomedical, Surgical and Dental Sciences of the Università degli Studi di Milano, located at the IRCCS Istituto Ortopedico Galeazzi, Milan, Italy, and from a private practice in Bristol, England. All subjects were treated following the principles included in the Helsinki Declaration and its further modifications [41]. The patients were exhaustively informed about the study protocol and the surgical procedures as well as the potential complications and drawbacks. After that, all patients signed a written consent form before entering the study.

### Patient selection

The following criteria were adopted for the selection of the cases:

#### Inclusion criteria

18 years of age or older with no general medical contraindications for oral surgical procedures (ASA-1 or ASA-2 according to the classification of the American Society of Anesthesiologists); all patients received a micro-surgical endodontics procedure of distal and/or mesial buccal roots of one or more upper molar teeth; absence of maxillary sinus sinusitis assessed by the absence of clinical signs (headache, rhinorrhea, altered taste, …) and by the external physical examination with palpation to observe any signs and symptoms triggered by finger pressure; be non-smokers or former smokers or smoke less than 10 cigarettes a day; tooth with a periradicular lesion of strictly endodontic origin (chronic apical periodontitis); tooth with non-surgical retreatment unfeasible or previously failed (post, anatomy, or iatrogenic complications); tooth with adequate final restoration without clinical evidence of coronal leakage; no spontaneous pain or swelling; good periodontal health condition at tooth level; be able to completely understand and sign an informed consent form.

#### Exclusion criteria

Presence of vertical root fracture; presence of root perforations; Miller class III/IV mobility; presence of root resorption; combined endodontic-periodontic lesions; pregnancy and patients with neuropsychiatric disorders; incomplete medical, tooth involved and sinus report; absence of preoperative and 3, 6, 12 months follow-up periapical radiograph; absence of preoperative CBCT; periapical lesion involving palatal root.

### Surgical procedure

All surgical procedures were performed by two oral surgeons between 2017 and 2019. Both surgeons have more than 10 years’ experience in endodontic surgery and have a periodontal surgery background.

Modern microsurgical techniques were performed using a surgical operating microscope (Zumax, 5 Zhiying street, Suzhou New District, China and Global, Global Surgical Corporation—3610 Tree Court Industrial Blvd.- Saint Louis, MO 63122) and surgical loupes. Before the procedure, a preoperative rinse with chlorhexidine digluconate (0.12%) was applied to reduce the number of bacteria on the surgical field.

Local anaesthesia with articaine 4% and epinephrine 1:100,000 was gently administered, avoiding blood vessels, deeply apical in the position of the affected tooth and the mesial and distal one. After that, a full thickness papilla preservation flap with two vertical incisions one from the vestibular aspect of the mesial tooth and the other from the distal aspect of the distal tooth or on the tuber was elevated and retracted carefully during the surgical procedure and continuously irrigated with sterile saline solution. Following flap elevation, when necessary, bone has been removed using round burs under irrigation to expose the root apex.

After the complete removal of the lesion, bone cavity has been carefully explored using magnification device for detection of a possible perforation of the maxillary sinus floor bone wall, with or without rupture of the exposed Schneiderian membrane. In case of perforation, a collage membrane was used as a shield in order to avoid a spread of endodontic material or root-tip inside the sinus.

Subsequently root apices were resected at least at 3 mm from the apex with a fluted fissure bur as perpendicular as possible (0°–15°) relative to the long axis of the root to eliminate all apical ramifications and lateral canals and to avoid reinfection of the periapical area and therefore the recurrence of the lesion.

The retrograde preparation of the root has been performed using ultrasonic microtips (Endo success apical surgery: AS3D, AS-lD, AS-RD, AS15LD, S12-70D, P15LD; Acteon Equipement (manufactured by Satelec)—17 avenue Gustave Eiffel, Zone Industrielle du Phare, BP 30216, 33708 MERIGNAC Cedex, France) in order to allow a parallel preparation. After adequate control of the haemostasis, the prepared root end was dried with paper points and sealed using mineral trioxide aggregate (OGNA-Aureoseal, Via Figini, 41, 20835 Muggiò MB, Italy and Endopass, DEI Italia srl., Via Torino 765 – 21020 – Mercallo, Varese, Italy). A surgical microscope was used for root end preparation in all patients. Finally, the flap was repositioned and sutured using polyamide 5–0 (Ethicon, Inc, Johnson & Johnson, Piscataway, NJ) with the aid of surgical loupes (4.5×).

Patients were advised to rinse twice daily with 15 mL chlorhexidine digluconate 0.2% up to 10 days after surgery. Anti-inflammatory nonsteroidal drugs (ibuprofen 400 mg) were prescribed to be consumed twice a day for two days after surgery for swelling and pain control. The prescription of antibiotics was avoided. Sutures were removed seven days after surgery.

### Radiographic evaluation

The distance of the most apical part of the roots (R) and of the lesion (L) from the floor of the maxillary sinus through the analysis of periapical radiograph and the CBCT was analysed under a value showed in Tables 1 and 2.

**Table 1:** Radiographic evaluation (root)

Relationship between the root and the maxillary sinus membrane	
Distance > 3 mm	R1
Distance < 3 mm but not in contact	R2
In contact with the maxillary sinus	R3
Inside the maxillary sinus	R4

**Table 2:** Radiographic evaluation (lesion)

Relationship between the lesion and the maxillary sinus membrane	
Distance > 3 mm	L1
Distance < 3 mm but not in contact	L2
In contact with the maxillary sinus	L3
Inside the maxillary sinus	L4

### Outcomes

The outcome was assessed 12 months after the surgeries based on radiographic and clinical evaluation following the criteria proposed by some studies [42–45], classifying the healing in:

Complete healing: include the absence of clinical signs/symptoms and the radiographic classification of complete or incomplete healing (clinical healing with a marked reduction in radiolucency).

Incomplete healing: include the absence of clinical signs/symptoms and the radiographic classification of uncertain healing. Persistence of radiolucency can be observed in the absence of clinical symptoms and signs, or the presence of clinical symptoms and signs associated with incomplete clinical recovery [42]. These cases must be monitored every 12 months for four years and if they remain doubtful after 4 years they are classified as failures [46].

Failure: include the presence of any clinical signs/symptoms such as pain, swelling, tenderness to percussion or palpation, or sinus tract and/or the radiographic classification of unsatisfactory healing.

All measurements were performed by evaluating the pre-operative CBCT scan and intraoral radiography done at 3, 6, 12 months after surgery. CBCT examinations were performed on a 3D Accuitomo XYZ Slice View Tomograph® (Model MCT-1, Type EX-1/EX-2; Fushimi-ku, Kyoto: J. Morita Mfg. Corp) with 60–80 kV and 1–10 mA, a voxel size of 0.125 mm per side, and an approximate exposure time of 18 s. Images were collected and analyzed using a computer software (OnDemand3D™, Cybermed, Seoul, South Korea), that was used to obtain all measurements.

The primary outcome was the clinical and radiographical success rate of endodontic surgery of upper molar teeth. The secondary outcomes were to assess a possible correlation of maxillary sinus floor perforation with or without rupture of the Schneiderian membrane during apical surgery of maxillary molars and the proximity of the treated root apices/periapical radiolucency as seen on CBCT and on periapical radiographs; to evaluate whether the injury of the Schneiderian membrane affects the outcome of the surgery and to assess one week after surgery the patient's quality of life.

A questionnaire was administered to evaluate postoperative limitations in function (mouth opening, chewing, speaking, sleeping, daily routine, and work) as well as pain and the presence of other symptoms (swelling, bleeding, nausea, bad taste/breath). The questionnaire structure was similar to that used in previous studies [19, 20, 36]. For pain assessment a VAS was adopted where 0 = no pain and 100 = the worst conceivable pain. For other symptoms and functional activities, the answers were based on a 5-point Likert-type scale, ranging from 0 (none) to 4 (very much). Finally, patients had to report whether they had taken any analgesics on each postoperative day. Patients were invited to fill out the questionnaire daily, starting on the day of surgery, for 7 days. Questionnaires were returned postage paid. Each questionnaire was coded and progressively numbered, so that the patient’s name did not appear. Thus, patients could not be identified.

### Statistical methods

Descriptive statistics was provided by calculating means and standard deviations for continuous variables. For categorical variables, the frequencies were computed and presented. One-way ANOVA was used to evaluate the diminishing of pain values over time.

The correlation between baseline parameters (the classification of the location of the periapical lesion, age, sex) and the outcomes was evaluated by means of logistic regression model. The same model served to calculate if the occurrence of any lesion of the maxillary sinus membrane has influenced the outcome.

The level of significance was *P* < 0.05.

## Results

A total of 35 patients were evaluated from the medical records of Dental Clinic of the Department of Biomedical, Surgical and Dental Sciences of the University of Milan, from the IRCCS Galeazzi Orthopaedic Institute and from a private practice in Bristol between 2017 and 2019. A total of 14 patients were excluded consequently 21 patients (Table 3) were included for a total of 27 roots and 29 canals treated (20 mesio-buccal (MB), 2 MB2 and 7 disto-buccal). Preoperative diagnostics consisted of a clinical examination, conventional periapical radiographs and CBCT. All 29 roots showed periapical radiolucency which indicated the presence of apical periodontitis.

**Table 3:** Demographic data of patients

Factors	IRCCS Galeazzi		Bristol		Total	
	n	%	n	%	n	%
*Sex*						
Male	8	57.1	4	57.1	12	57.1
Female	6	42.9	3	42.9	9	42.9
*Age*						
> 45	6	42.9	1	14.3	7	33.3
< 45	8	57.1	6	85.7	14	67.7
*Tooth type*						
1.6	5	35.7	2	28.6	7	33.3
1.7	1	7.2	0		1	4.8
2.6	6	42.8	5	71.4	11	52.4
2.7	2	14.3	0		2	9.5
*Smoker*						
Yes	4	28.6	1	14.3	5	23.8
No	10	71.4	6	85.7	16	76.2
*Fistula*						
Yes	0		5	71.4	5	23.8
No	14	100	2	28.6	16	76.2

Of this patients, 14 undergone microsurgical endodontics at the IRCCS Galeazzi Orthopaedic Institute of Milan, average aged 48 years (range 38–72) 6 females and 8 males and 7 from Bristol (private practice) average aged 55 years (range 40–71) 3 females and 4 males. In total, the sample consisted of 42.9% female and 57.1% male with an average age corresponding to 51.5 years. The most frequently treated tooth corresponds to maxillary left first molar (52.4%, 11 cases) followed by maxillary right first molar (33.3%, 7 cases), maxillary left second molar (9.5%, 2 cases) and maxillary right second molar (4.8%, 1 case). At the recruitment only five patients on 21 presented with a gingival fistula (Table 3). All treated patients were ASA 1.

In six cases the maxillary sinus membrane has been damaged and only in one case it required to be repaired with a collagen membrane.

With regard to the success of the treatment, 13 patients were in group 1 (success healing) and 8 in group 2 (uncertain healing) both at 3 and 6 months. At 12 months 18 patients were in group 1 (success healing) and only three in group 2 (uncertain healing). The success rate in this clinical study was 85.7% after one year. In 28.5% (6 patients) there was a perforation of the Schneiderian membrane, which was repaired only in one case, and in five cases the patients experienced nose bleeding, during the night after surgery and the following day. Of these six treated elements belonging to subjects that experienced complications, one had roots with a distance of more than 3 mm from the maxillary sinus (R1), while three had roots closer than 3 mm to the maxillary sinus (R2) and two were in contact with the maxillary sinus floor (R3); the lesion in only one case was not in contact with the maxillary sinus (L2) while in two cases it was in contact with the maxillary sinus (L3) and in three it was inside the maxillary sinus (L4). Of these patients, two at the radiographic control at 1 year were in group 2 (Uncertain healing) while the other four were completed healed. 100% of patients kept the molar 1 year later. None of these 21 patients had an alteration of the physiology of the maxillary sinus.

**Fig. 1:**
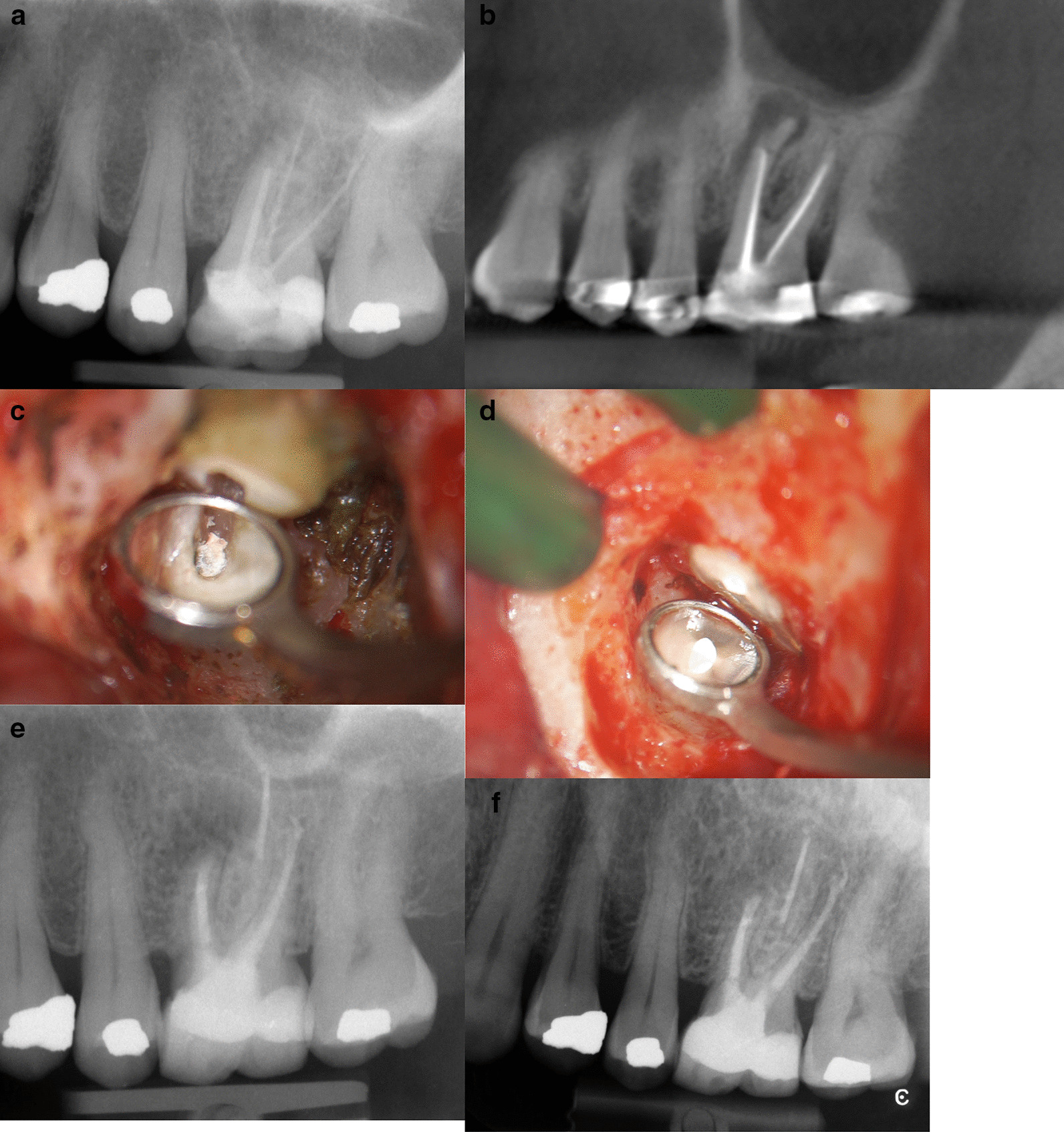
**a** Pre-operative periapical radiograph showing perforation on MB root and apical lesion. Classified R1-L2. **b** Pre-operative CBCT showing a different classification Classified R2-L3. **c** This microscopic picture highlights the root-end resected and the incomplete filling of the root canal system. **d** Picture showing root-end filling. **e** Periapical radiograph at 6 months follow-up. After Microsurgical Endodontics on MB ROOT showing an incomplete healing. **f** Periapical radiograph at 12 months follow-up showing a complete healing

**Fig. 2:**
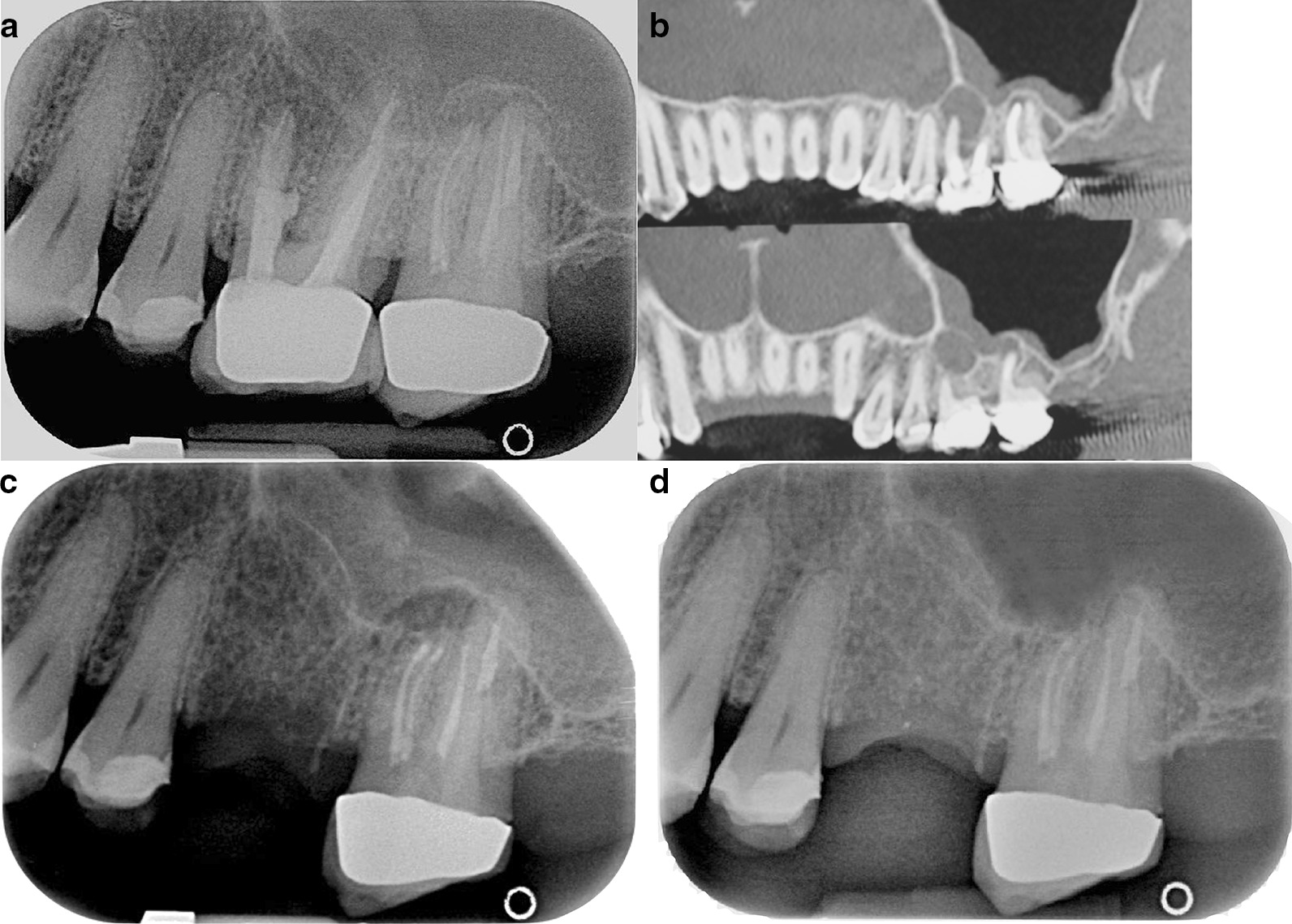
**a** Pre-operative periapical radiograph showing perforation on MB of 2.6 and a 2.7 periapical lesion of MB and DB. Classified: 2.6: R2-L3 and 2.7: R3-L4. Tooth 2.7 was treated 21 months ago and subsequently retreated 12 months ago. It is evident that it is difficult to distinguish exactly which roots are affected by tooth pathology 2.7 using a 2D image. **b** Pre-operative CBCT showing a different classification. 2.6 Classified R3-L4 and 2.7 classified R4 and L4. **c** Periapical radiograph at 6 months follow-up. After Microsurgical Endodontics on MB1 e MB2 and DB root of 2.7 showing an incomplete healing. Tooth 2.6 was extracted. **d** Periapical radiograph at 12 months follow-up. After Microsurgical Endodontics on MB1 e MB2 and DB root of 2.7 showing a complete healing. Tooth 2.6 was extracted

**Fig. 3:**
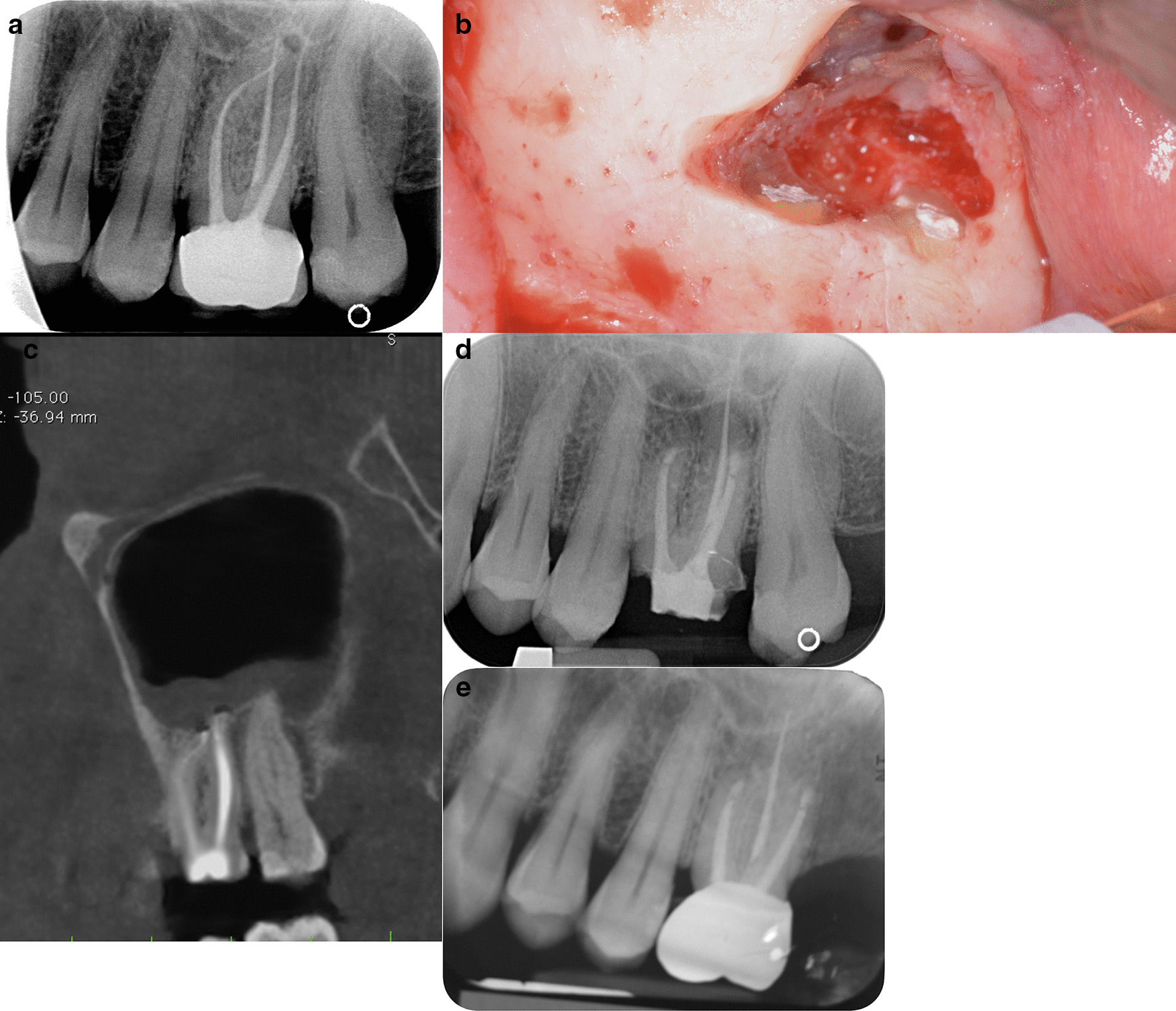
**a** Pre-operative periapical radiograph of 2.6 showing a small apical lesion of MB root. Classified R3-L3. **b** Mesial and distal buccal root showing root-end filling. A small perforation of the sinus membrane is evident above the roots. **c** Pre-operative CBCT showing both MB and DB with a periapical lesion inside the sinus (R3, L4) and a thickness of the Schneiderian membrane. **d** Periapical radiograph at 6 months follow-up. After Microsurgical Endodontics on MB and DB root of 2.6 showing an incomplete healing. Tooth 2.6 have a resin temporary crown. **e** Periapical radiograph at 12 months follow-up. After Microsurgical Endodontics on MB and DB root of 2.6 showing a complete healing. Tooth 2.6 have metal-ceramic crown

Figures 1a–f, 2a–d and 3a–e showed three clinical cases.

**Fig. 4:**
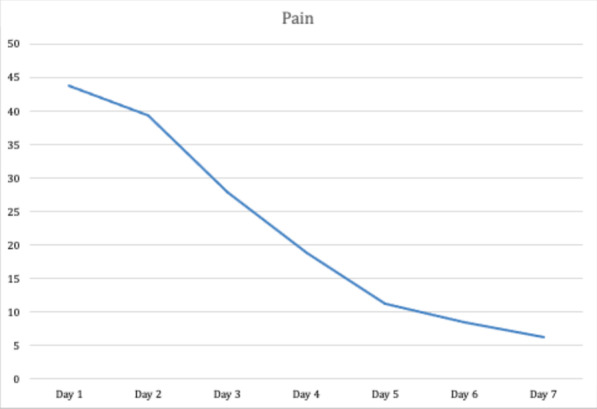
Trend of pain levels over time during the first week after surgery

The pain level decreased significantly over time and the trend is represented in Fig. 4. The impact of the intervention on the items considered in the questionnaire was negligible.

## Discussion

The aim of the present study was to evaluate if endodontic surgery of the upper molars was a predictable technique that could give a good success rate and if the proximity of the maxillary sinus to the lesion or roots and a perforation of the Schneider membrane could influence the outcome of the intervention. In the literature, few studies have focused on endodontic surgery of upper molars. In general, it has been seen that success ranges from 44 to 88% [21, 40]. In our study we have seen that this surgical technique has a good success rate (85.7%). The difference in success may be due to the fact that conventional technique was used in the past with greater difficulty in locating the lesion, cleaning and obturating the apical part of the root system [47], while now with the use of microsurgical instruments, ultrasonic retro-tips, biocompatible root-end filling material and therefore the use of magnification systems that allow a more reduced osteotomy, a resection of the apex with a minimum or zero resection angle, specific ultrasonic tips for the instrumentation of the retrograde cavity and highly biocompatible filling materials the surgery has become more efficient with less chance of an error [47–49].

A further question that should be addressed is whether a perforation of the Schneiderian membrane with the formation of an oro-antral communication (OAC) is to be considered a "surgical accident" [33] or if it is to be accepted as an accident less likely to occur in periapical surgery of maxillary posterior teeth considering the close relationship with the maxillary sinus.

The low number of postoperative complications in our cases with OAC tells us that it is a possible complication that does not interfere with the healing process and therefore the success of the surgery. In nine of the six patients with membrane perforation, we didn’t find specific postoperative adverse events. Although it should be emphasised that we had nosebleeds in five cases, that resolved spontaneously within 24 h. At 1 year follow-up of three patients with uncertain healing two had perforation. This may be due, in our opinion, to the fact that healing in patients with roots in contact with the maxillary sinus could be slower and less visible from the x-ray because it is more difficult for a clot to form. These cases will be monitored for 4 years at the end of which they will be included in the group of “success healing” or in the “failed” group.

A recent literature review article on periapical surgery by Garcia et al. [50] confirms this finding: none of the included studies found a significant difference in healing outcome or postoperative sequelae when accidental OAC occurred.

Notwithstanding the above, foreign material, drilling dust or bacteria should be prevented from entering the sinus during surgical procedures. Surgical magnification aids such as magnifying glasses, endoscopes and microscopes allow the surgeon to make an accurate surgical diagnosis and above all, they can enable better control of surgical acts by preventing undesirable situations such as those mentioned above, making this surgery more controllable and therefore safer.

Previous clinical studies have reported maxillary sinus perforation in conjunction with apical surgery in the range of 10.4–50.0% [21]. Possible explanations of this difference in percent could be the difference technique proposed.

An aspect to be taken into account is what in the past the operation could be could also be indicated and thus executed. in a state of subacute or acute infection; the close relationship between the roots and a compromised maxillary sinus (eg polyposis, swelling of the Schneiderian membrane, chronic sinusitis) due to foreign bodies observed preoperatively; the use of amalgam as a retrograde filling material; the dimensions of the handpieces for the preparation of retrograde cavities for amalgam are larger than those of the sonic / ultrasonic retro-tips currently used. Therefore, periapical surgery using the conventional technique requires more periapical space and creates a larger osteotomy defect. In our study, the percentage of perforation (28.5%) is higher than in other studies (Oberli 9.6%; Friedman et al. 11.8%) but probably because the other studies do not focus only on molars where there is a closer relation between the roots and the maxillary sinus.

The surgical instruments used in our study for the ostectomy and the access to the apical part of the root and the lesion consisted in a fine-grain diamond burs but, as an alternative, it can be considered the use of piezoelectric handpiece with specific insert [51]. Future randomized clinical studies may indicate if there is a difference using one approach or the other with regard to this specific surgery.

We have also noticed that the most easily bias on this type of study is the performance bias, an error that depends on the fact that who performed this type of surgery was an expert operator. Other limitations of this study are that endodontic surgery of the upper molar tooth is a difficult surgery that requires not only experienced operators in the field of oral surgery where knowledge of anatomy is fundamental but also in periodontology for the preservation of soft tissues and endodontics for retrograde obturation; that requires very specific dedicated materials, such as microsurgical instruments and magnification systems; it is necessary to perform a CBCT to study the anatomy of the element to be treated, the size of the lesion and the proximity to the maxillary sinus in order to plan the intervention in the best possible way. Eberhardt et al. [23] stated that "standard dental radiographs, including orthopantomography, present a two-dimensional image and as such are inadequate and/or impractical for a precise morphometric assessment of bone relationships." There are often important discrepancies between what can be observed from intraoral radiographs and CBCT.

As a manner of fact, we also noticed many discrepancies from what we observed from the intraoral radiograph and CBCT scans: for example, while only in one patient the lesion seemed inside the maxillary sinus (L4) on the apical X-ray, in reality five patients were in L4 group at the analysis of the CBCT.

To our knowledge there are a limited number of studies that specifically assess the success of endodontic upper molar surgery. Different cross-sectional studies [52–55] have reported that the prevalence of apical periodontitis and other post-treatment periapical disease can exceed 30% of a full-filled teeth population. Endodontic nonsurgical retreatment planning must include a careful evaluation of the teeth features, so a decision can be made among non-surgical re-treatment, surgical (retrograde) surgery or tooth extraction [56].

Therefore, in case of a tooth with a periradicular lesion of strictly endodontic origin following failed endodontic therapy or when nonsurgical treatment is not feasible or not recommended, endodontic surgery has the objective of preserving the dental element.and give to the patient the less invasive, less expensive and shorter treatment.

The therapeutic alternative would be less conservative, and would include the extraction of the compromised tooth with the positioning of an implant or the execution of a traditional prosthesis. In the first case, due to the presence of the maxillary sinus, the treatment often needs interventions to increase the amount of the available bone, such as sinus floor elevation. Many studies have also demonstrated that teeth even compromised because of endodontic problems may have a longevity that surpasses the average implant and preserve the natural dentition should be the first choice [57–59].

## Conclusion

In conclusion, despite the limitations presented, the success of the endodontic surgery of the upper molar teeth can be considered a valid treatment option and we can therefore draw some considerations: it is a predictable technique that has a good success rate if it is performed by experienced operators with the use of appropriate tools. The perforation of the Schneiderian membrane does not seem to be a factor that influence the success of the surgery and/or the quality of life of the patient. Preoperative CBCT Scan provides significant information about the location of the roots and of the lesions and their relationship to the maxillary sinus.

Further studies with larger sample size and which also focus on the treatment of the palatal root are needed to compare the results obtained.

## Data Availability

The datasets used and analyzed during the current study are available from the corresponding author on reasonable request.
